# Supplementary material for: HIVprotI: an integrated web based platform for prediction and design of HIV proteins inhibitors
Source: J Cheminform. 2018 Mar 9;10:12. doi: 10.1186/s13321-018-0266-y (PMC5845081; doi:10.1186/s13321-018-0266-y)
Supplement: Supplementary file 2 — Additional file 2. Source code of HIVProtI web server. [file 13321_2018_266_MOESM2_ESM.zip › HIVprotI_Source-code/hivprotI/weka-3-6-6/doc/weka/classifiers/Sourcable.html]

Sourcable


---


|  |  |  |
| --- | --- | --- |
| **Overview** \| **Package** \| **Class** \| **Tree** \| **Deprecated** \| **Index** \| **Help** \| **Weka's home** | |  |
| **PREV CLASS**   **NEXT CLASS** | **FRAMES**    **NO FRAMES**     **All Classes** |  |
| SUMMARY: NESTED \| FIELD \| CONSTR \| METHOD | DETAIL: FIELD \| CONSTR \| METHOD |  |


---


## weka.classifiers Interface Sourcable

**All Known Implementing Classes:**: AdaBoostM1, DecisionStump, Id3, J48, J48graft, LogitBoost, MultiBoostAB, OneR, REPTree, ZeroR

---

``` public interface Sourcable ```

Interface for classifiers that can be converted to Java source.

**Version:**
:   $Revision: 1.6 $

**Author:**
:   Len Trigg (trigg@cs.waikato.ac.nz)

---

| **Method Summary** | |
| --- | --- |
| `java.lang.String` | `toSource(java.lang.String className)`             Returns a string that describes the classifier as source. |

**Method Detail**

### toSource

```
java.lang.String toSource(java.lang.String className)
                          throws java.lang.Exception
```

:   Returns a string that describes the classifier as source. The
    classifier will be contained in a class with the given name (there may
    be auxiliary classes),
    and will contain a method with the signature:

    ```
     public static double classify(Object [] i);
    ```

    where the array `i` contains elements that are either
    Double, String, with missing values represented as null. The generated
    code is public domain and comes with no warranty.

    :   **Parameters:**: `className` - the name that should be given to the source class. **Returns:**: the object source described by a string **Throws:**: `java.lang.Exception` - if the source can't be computed


---


|  |  |  |
| --- | --- | --- |
| **Overview** \| **Package** \| **Class** \| **Tree** \| **Deprecated** \| **Index** \| **Help** \| **Weka's home** | |  |
| **PREV CLASS**   **NEXT CLASS** | **FRAMES**    **NO FRAMES**     **All Classes** |  |
| SUMMARY: NESTED \| FIELD \| CONSTR \| METHOD | DETAIL: FIELD \| CONSTR \| METHOD |  |


---
